# Dietary patterns influencing the human colonic microbiota from infancy to centenarian age: a narrative review

**DOI:** 10.3389/fnut.2025.1591341

**Published:** 2025-06-04

**Authors:** Vitor Geniselli da Silva, Nicole Clémence Roy, Nick William Smith, Clare Wall, Jane Adair Mullaney, Warren Charles McNabb

**Affiliations:** ^1^Riddet Institute, Massey University, Palmerston North, New Zealand; ^2^High-Value Nutrition National Science Challenge, Auckland, New Zealand; ^3^Department of Human Nutrition, University of Otago, Dunedin, New Zealand; ^4^Department of Nutrition and Dietetics, The University of Auckland, Auckland, New Zealand; ^5^AgResearch, Palmerston North, New Zealand

**Keywords:** gut microbiota, diet, infant, adult, older adult, centenarian

## Abstract

Our dietary choices not only affect our body but also shape the microbial community inhabiting our large intestine. The colonic microbiota strongly influences our physiology, playing a crucial role in both disease prevention and development. Hence, dietary strategies to modulate colonic microbes have gained notable attention. However, most diet-colonic microbiota research has focused on adults, often neglecting other key life stages, such as infancy and older adulthood. In this narrative review, we explore the impact of various dietary patterns on the colonic microbiota from early infancy to centenarian age, aiming to identify age-specific diets promoting health and well-being by nourishing the microbiota. Diversified diets rich in fruits, vegetables, and whole grains, along with daily consumption of fermented foods, and moderate amounts of fish and lean meats (two to four times a week), increase colonic microbial diversity, the abundance of saccharolytic taxa, and the production of beneficial microbial metabolites. Most of the current knowledge of diet-microbiota interactions is limited to studies using fecal samples as a proxy. Future directions in colonic microbiota research include personalized *in silico* simulations to predict the impact of diets on colonic microbes. Complementary to traditional methodologies, modeling has the potential to reduce the costs of colonic microbiota investigations, accelerate our understanding of diet-microbiota interactions, and contribute to the advancement of personalized nutrition across various life stages.

## 1 Introduction

The human gastrointestinal tract hosts a diverse and dynamic microbial community, including bacteria, archaea, fungi, and viruses, which play key roles in host health and wellbeing. Most microbes are found in the large intestine or colon, with an estimated concentration of 10^11^ cells/mL (1), although colonic microbial abundance can vary depending on the sample site, analytical method used, and host physiology. Fecal samples are non-invasive proxies to study the relationship between colonic microbes and human health, particularly microbial composition and function. These analyses have revealed the impact of colonic commensals on host nutrition, metabolism, and the immune and neurological systems (2–8). However, many crucial aspects of this relationship remain unknown. Defining what constitutes a healthy colonic microbiota composition and function is an ongoing challenge (9, 10).

One of the major challenges in investigating the colonic microbiota is its substantial compositional variability, which occurs both within the same individual over time and between individuals (11, 12). This variation limits predicting how each individual’s microbiota may respond to interventions and their subsequent impact on host health. Colonic microbes interact with one another and the host, forming a dynamic network influenced by various individual and environmental factors. These factors mainly include dietary habits, host health status, genetics, age, gender, geographical location, lifestyle behaviors, and antibiotic use (13).

Among the factors under host control, diet is key. Dietary compounds not absorbed by the small intestine reach the colon, are fermented by colonic microbes, and produce metabolites that influence host physiology. Dietary interventions can rapidly alter the composition and function of the colonic microbiota (14). For instance, non-digestible carbohydrates have prebiotic properties. Their consumption promotes the growth of saccharolytic microorganisms and increases the production of beneficial short-chain fatty acids (SCFAs) (15, 16). In contrast, a diet lacking non-digestible carbohydrates and with excessive intake of protein and fat from animal origin increases the abundance of pathogens and the production of potentially deleterious molecules (17–19). Recently, phytochemicals, bioactive compounds found in plants, have attracted attention for their potential prebiotic effect, as well as antioxidant and anti-inflammatory properties (20).

However, the long-term effect of dietary patterns on colonic microbes remains unclear, including the persistence of diet-induced alterations in the colonic microbiota when changing dietary exposures. Furthermore, diet-colonic microbiota research has predominantly focused on adults (ages 18-65), limiting our understanding of how diet influences colonic microbes in other life stages. Notably, dietary patterns during infancy (under 3 years) and older adulthood (over 65 years) differ from those in adulthood, likely influencing the composition and function of the colonic microbiota and highlighting the need for more investigations specific to these age groups (21, 22). Literature reviews on the influence of diet on colonic microbiota across the human lifespan are scarce and often do not fully cover all life stages (23, 24). Considering these knowledge gaps, this narrative review examines how different dietary patterns influence the human colonic microbiota across early infancy, weaning, adulthood, older adulthood, and centenarian age. This review aims to identify age-appropriate diets for microbiota modulation, providing insights into promoting health and wellbeing.

## 2 Search strategy

A narrative review was conducted to evaluate the impact of dietary patterns on the human colonic microbiota across infancy (under 3 years), adulthood (between 18 and 65 years), older adulthood (between 65 and 100 years), and centenarian age (over 100 years). Articles were primarily identified through searches in the PubMed and Google Scholar databases using the terms “gut microbiota”, “diet”, and “infant OR adult OR older adult OR centenarian”. Additional records evaluating the effect of dietary patterns on the colonic microbiota were identified by replacing “diet” with terms referring to specific dietary patterns (e.g., “gut microbiota” AND “western diet” AND “infant OR adult OR older adult OR centenarian”). Only studies involving humans and published in English were identified. No specific inclusion and exclusion criteria were applied. Articles were prioritized based on their publication date, giving preference to the most recent studies. When available, meta-analyses, systematic reviews, and randomized controlled trials were prioritized.

## 3 Principal colonic microbes and produced microbial metabolites

The colonic microbiota is dynamic, and its composition varies throughout life. Its development is proposed to have an initial phase until the first 14 months of postnatal life, followed by a transitional period between 15 and 30 months, reaching stability after 31 months (25). Multiple factors influence the composition and function of colonic commensals over life, resulting in a unique profile of colonic microbes for each individual (13, 25). Despite this variability, certain taxa and gene functions prevail in healthy adults based on fecal data, suggesting the existence of core microbial functional groups (26, 27). The principal microbial taxa in the colonic microbiota and their associated microbial metabolites are summarized in Supplementary Tables 1, 2, respectively.

To date, the mechanisms by which colonic microbes influence host health remain unclear. Under favorable conditions, colonic microbes may support host homeostasis by competing with pathogens for resources, producing anti-microbial metabolites, and modulating host immune responses (28, 29). Conversely, disruptions in the colonic microbiota can favor the growth of pathogens and the production of pro-inflammatory metabolites, compromising the integrity of the colonic epithelial barrier. This allows luminal molecules and microbes to enter the bloodstream, potentially triggering excessive host immune responses. Over time, these responses may lead to a chronic inflammatory state and increased disease risk. Additionally, it is plausible that certain diseases may alter the colonic environment, disrupting the colonic microbiota and creating a vicious cycle that perpetuates disease.

## 4 Influence of dietary patterns on colonic microbes

### 4.1 Infancy

During the first months of postnatal life, the colonic microbiota composition is mainly affected by the gestational age, mode of delivery, and type of feeding (human milk versus infant formula) (25, 30, 31). Systematic literature reviews suggested that full-term pregnancy, natural birth, and breastfeeding provide greater opportunities for the infant colonic microbiota to thrive (32, 33). In contrast, pre-term pregnancy, C-section, and formula-feeding are associated with disruptions of the microbial community (25, 31). In regards to feeding in early infancy, breast milk contains oligosaccharides that promote the growth of the genus *Bifidobacterium* and support beneficial microbial cross-feeding interactions (34, 35). In addition, breast milk is not sterile and contains microbes that can colonize the infant’s colon, as well as bioactive compounds like antibodies and lactoferrin, which reduce pathogen colonization (36, 37).

In contrast, infant formulas are predominantly made with bovine milk, have higher protein content, and are often supplemented with fructooligosaccharides and galactooligosaccharides to provide prebiotic effects. While infant formulas meet the nutritional requirements for infant development, they fail to mimic the bifidogenic effect of human milk. Systematic reviews have found that, compared to breastfed infants, formula-fed infants exhibit a lower fecal abundance of the genus *Bifidobacterium*, an increased abundance of pathogens, and a higher expression of microbial genes associated with amino acid metabolism (38, 39).

**FIGURE 1:**
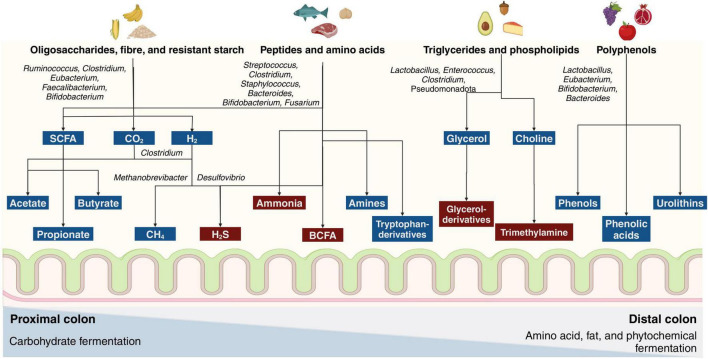
Fermentation of dietary compounds by the colonic microbiota. Metabolites in blue benefit the host, while those in red are potentially deleterious. A decrease in carbohydrate fermentation is observed from the proximal to the distal colon, mainly producing short-chain fatty acids (SCFAs) and gases. On the other hand, amino acid, fat, and phytochemical fermentation increase from the proximal to the distal colon. Microbial fermentation of amino acids produces SCFAs, branched-chain fatty acids (BCFAs), biogenic amines, hydrogen sulfide (H_2_S), and ammonia. Fermentation of dietary fats is involved in trimethylamine production, whereas the fermentation of polyphenols leads to the generation of multiple bioactive molecules.

Exclusive breastfeeding is recommended for the first 6 months of life (40). Nutrients from complementary foods reaching the colon unabsorbed mature the infant’s colonic microbiota toward a more adult-like configuration (Table 1). Non-digestible carbohydrates are preferentially fermented by colonic microbes, producing SCFAs and gases (Supplementary Table 2), and their availability in the colon limits the fermentation of other dietary compounds (41, 42). *In vitro* evidence suggests that carbohydrate fermentation primarily occurs in the proximal colon, while other macronutrients are metabolized in the distal colon (43, 44) (Figure 1). Notably, SCFAs are molecules that exert well-documented health benefits, and their reduced fecal levels are frequently observed in preterm infants, adults with autoimmune, metabolic, and gastrointestinal diseases, and older adults with neurological disorders (15, 45–48). However, excessive fermentation of rapidly fermented fibers can increase gas production in individuals with functional gastrointestinal disorders, leading to bloating, pain, and discomfort (49).

**TABLE 1:** Impact of complementary foods on the colonic microbiota of weaning infants.

Food category	Food intervention trials	Observational studies	*In vitro* fecal fermentations	References
Meats (e.g., beef, pork)	↑Clostridiales, *Clostridium XIVa* ↓*Enterobacteriaceae* ↑alpha diversity (Chao1 and Shannon indexes)	↓*Bacteroides* ↑alpha diversity (Shannon index)	Not evaluated	(72, 82, 84, 85, 94, 95)
Dairy (e.g., yogurt, cheese)	Not evaluated	↓*Bacteroides, Clostridiaceae* ↑alpha diversity (Shannon index)	↑*Bifidobacterium, Lactobacillus, Enterococcaceae* ↓*Enterobacteriaceae* ↑acetate, propionate, butyrate	(72, 84, 88, 96)
Infant cereals (e.g., whole-grain and refined cereals)	↑Bacteroidales, *Bacteroides* ↓*Enterobacteriaceae*, *Escherichia-Shigella*	↑alpha diversity (richness and Shannon index)	↑*Bacteroidaceae, Prevotellaceae, Ruminococcaceae* ↑acetate	(81–83, 85, 86)
Fruits and vegetables (e.g., apple, berries, carrot)	Not evaluated	↑alpha diversity (Shannon index)	↑*Bifidobacterium, Lactobacillus, Streptococcus, Ruminococcus, Faecalibacterium* ↓*Clostridium, Enterobacteriaceae* ↑acetate, propionate	(83, 87, 88)
Sweets (e.g., cakes, desserts, chocolates)	Not evaluated	↓*Bifidobacterium, Clostridium cluster IV*	Not evaluated	(50)

Longitudinal evidence demonstrated that colonic microbial diversity and stability increase during weaning, and genes associated with carbohydrate degradation, vitamin biosynthesis, and production of SCFAs are enriched (50). The abundances of the genera *Bifidobacterium* and *Lactobacillus* decrease, while the phyla Bacillota and Bacteroidota and the genera *Clostridium* and *Bacteroides* are enriched (50–52). Furthermore, the dominant fungal species *Debaryomyces hansenii* is replaced by *Saccharomyces cerevisiae* and the viral richness and diversity decrease (53, 54).

Diet-induced microbiota changes during infancy may have long-lasting effects, influencing susceptibility to diseases later in life (55–57). Weaning is crucial for the colonic microbiota development, influencing health and wellbeing later in life. Although many studies have characterized the influence of feeding type (breastmilk versus infant formula) on the development of the infant gut microbiota (25, 31, 38, 39), the impact of complementary foods on colonic microbes of weaning infants remains underexplored. A recent systematic review of interventional trials assessing the effects of complementary foods on fecal microbiota composition in weaning infants found that whole-grain cereals and meats increased the fecal abundance of SCFA-producing bacterial taxa and increased microbial richness (58). However, the review was limited to only seven clinical trials characterizing the microbiota using 16S rRNA gene sequencing, highlighting the scarcity of food interventions in this field.

Another notable knowledge gap is the limited understanding of how maternal diet influences the infant’s colonic microbiota. Maternal intake of plant-based dietary compounds during pregnancy has been reported to influence the neonatal fecal microbiota (59), while increased maternal dietary diversity and consumption of fermented foods during pregnancy were associated with lower fecal alpha diversity in infants (60). Additionally, the mother’s diet affects the microbial and nutritional composition of breastmilk, which may impact colonic microbes of breastfed infants (61). A scoping review identified associations between maternal dietary patterns and infant fecal microbiota composition. Maternal consumption of seafood, fermented dairy, fruits and veggies, and nuts was linked to an increased abundance of beneficial taxa in the infant fecal microbiota (33). Conversely, maternal intake of artificial sweeteners and high-fat diets were associated with negative alterations in the infant microbiota (33).

Artificial sweeteners, along with emulsifiers, thickeners, stabilizers, preservatives, and colorants, are chemicals added during food production to extend shelf-life or modify sensory properties. Although little is known about the effect of food additives on colonic microbes, a systematic review of randomized controlled trials, encompassing participants from infants to older adults, found that maltodextrin impacts the composition of the fecal microbiota, notably the abundance of the genera *Lactobacillus* and *Bifidobacterium* (62). Furthermore, another systematic review, including *in vivo* and *in vitro* studies, reported that exposure to colorants, sweeteners, emulsifiers, and preservatives is linked with perturbations in the colonic microbiota and adverse health effects (63). These findings highlight the need for further research and support recommendations to reduce the consumption of ultra-processed foods (64).

Another topic that requires further research is the interplay between micronutrients and the infant colonic microbiota. Vitamins are mostly absorbed in the upper small intestine, while minerals typically have lower bioavailability and reach the colon in greater amounts (65). Colonic microbes influence micronutrient levels by synthesizing vitamins and modulating mineral absorption (66, 67). In turn, micronutrients affect their composition and function. For instance, a systematic review of observational human studies reported that vitamin B12 supplementation may increase the alpha diversity of the fecal microbiota in adults and older adults but not in infants (68). In contrast, iron intervention supplementation in infants, commonly used to combat malnutrition, may increase the fecal abundance of pathogens, as observed in randomized controlled trials profiling the microbiota using 16S rRNA gene sequencing (69, 70).

Current nutritional guidelines recommend introducing infants to a diverse range of complementary foods (64, 71). Consistently, fecal microbiota observational studies have shown that greater dietary diversity during weaning is associated with increased microbial diversity and richness (72, 73). Importantly, dietary diversity in infancy is essential to prevent nutrient deficiencies often linked with monotonous diets, which can contribute to perturbations in the colonic microbiota and subsequent elevated risk of disease (74). In line with these recommendations, various fruits, vegetables, and whole grains are recommended for weaning infants (64, 71, 75). These foods are sources of vitamins, minerals, complex carbohydrates and phytochemicals, leading to beneficial alterations in colonic microbes (Table 1). Phytochemicals have recently attracted attention in colonic microbiota research for their ability to support the growth of beneficial taxa and inhibit pathogens (76), in addition to having anti-inflammatory and antioxidant properties. Polyphenols are the most prominent phytochemicals, with less than 10% absorbed in the small intestine and the majority reaching the distal colon, where they may be converted into more bioactive and bioavailable molecules by colonic microbes (77, 78). This microbial conversion is associated with various health benefits to the host, such as cardioprotective and antimetabolic syndrome effects, as observed in adults (79, 80).

Whole-grain infant cereal interventions in weaning infants increased the fecal abundance of SCFAs-producing bacteria, such as the genus *Bacteroides*, while reducing the abundance of pathogens belonging to the family *Enterobacteriaceae*, as determined using 16S rRNA gene sequencing (81, 82). Consistently, observational trials profiling the fecal microbiota of weaning infants by 16S rRNA sequencing, positively correlated the intake of complex carbohydrates with increased fecal alpha diversity and abundance of *Lachnospiraceae* and *Ruminococcaceae* families (83, 84). These findings were further confirmed by a longitudinal trial that used shotgun metagenomic sequencing to evaluate the composition of the infant fecal microbiota during the first year of life. The study highlighted the key role of complex carbohydrates in supporting the maturation of the colonic microbiota in weaning infants, as evidenced by an increased fecal alpha diversity and abundance of SCFA-producing bacterial genera (85). *In vitro* fecal fermentations using inoculum from weaning infants reported that whole grain cereals and numerous fruits and vegetables promoted the growth of the *Ruminococcaceae* and *Prevotellaceae* families to the detriment of the *Enterobacteriaceae* family, leading to acetate production after 24 h of fermentation (86–88). An *in silico* investigation recently identified berries as promising candidates for increasing the production of acetate and propionate by the infant’s fecal microbiota when consumed with breastmilk (89). Similarly, a 24-h *in vitro* fermentation study using weaning infant inoculum found that berries increased acetate and propionate production (90).

Nutritional guidelines also recommend moderate consumption (one serving per day) of lean meats and fermented dairy for infants to meet protein and micronutrient requirements (64, 71, 75). Introducing protein-rich foods to infants increased their colonic availability of amino acids, as their protein digestion and absorption are not yet fully developed (91). In the colon, the fermentation of unabsorbed amino acids and small peptides produces SCFAs (30% of protein mass), branched-chain fatty acids (BCFAs), tryptophan-derivatives, and biogenic amines, but also pro-inflammatory metabolites, such as hydrogen sulfide (H_2_S) and nitrogen-derivatives (92, 93). Consistently, clinical trials demonstrated that introducing protein-rich foods to weaning infants increases their fecal abundance of SCFA-producing taxa. For instance, pureed beef interventions in infants increased fecal abundance of *Clostridium XIVa* members, reduced the abundance of the *Enterobacteriaceae* family, and increased alpha diversity, as determined using 16S rRNA sequencing (82, 94, 95). Similarly, an observational study profiling the infant fecal microbiota using 16S rRNA amplicon sequencing reported a positive association between meat intake and increased fecal alpha diversity (72). For dairy products, their consumption by weaning infants was negatively associated with the abundance of the *Bacteroides* genus and the *Clostridiaceae* family, according to an observational study that profiled the fecal microbiota of infants aged 6-24 months using 16S rRNA gene sequencing (96). In turn, the fermentation of bovine milk for 10 h using feces from infants at weaning age increased the abundance of taxa from the *Bifidobacterium* genus *in vitro* (88).

An observational trial profiling the microbiota of weaning infants by 16S rRNA amplicon sequencing reported that dietary patterns characterized by the consumption of foods rich in protein and in fiber-rich foods, such as meats, cheese, and wholegrain bread, have been positively correlated with the increased fecal abundance of taxa from the Lachnospiraceae family, decreased abundance of taxa from the Bifidobacteriaceae family, and increased microbial alpha diversity (72). On the other hand, the consumption of diets rich in fat but low in carbohydrates has been associated with reduced fecal levels of SCFAs and increased abundance of pathogens in post-weaning infants (97). Dietary fats are normally well-digested and absorbed, with less than 5% of ingested fat reaching the colon (98). Importantly, their influence on colonic microbes depends on their type: as concluded by a systematic review of adult studies, saturated fatty acids are associated with reduced colonic microbiota richness and diversity, whereas polyunsaturated fatty acids do not exhibit this effect (99). Furthermore, certain fatty acids have anti-microbial activity and can reduce the abundance of pathogenic taxa (100). In this context, the Mediterranean diet, rich in polyunsaturated fatty acids from olive oil, was recently adapted to children (aged 3 and older) to help prevent obesity and cardiometabolic diseases in infancy and later life. The adapted diet emphasizes the daily consumption of fruits, vegetables, legumes, nuts, whole grains, olive oil, and dairy, along with weekly consumption of fish, eggs, and meats (101).

### 4.2 Adulthood

The complete maturation of the colonic microbiota is believed to occur around 3 years old (102). Nevertheless, evidence suggests its continued functional and compositional development during childhood and adolescence (103, 104). Compared to other life stages, more is known about the influence of dietary patterns on the colonic microbiota of adults. This topic has been extensively reviewed in recent publications (105–107). Therefore, only the impact of common diets on the colonic microbes of adults is briefly discussed here (Table 2).

**TABLE 2:** Impact of various dietary patterns on the adult colonic microbiota.

Diet	Definition	Microbial composition	Microbial function	Observed effect of diet on host health	References
Western diet	High intake of protein, fat, and sugars. Low consumption of complex carbohydrates	↑*Bacteroides, Alistipes, Penicillium* ↓*Methanobrevibacter*	↑Amino acid and bile acid metabolism and production of nitrogen derivatives and BCFAs	Adherence to a Western diet increased risk factors for metabolic and cardiovascular diseases compared to a prudent dietary pattern	(108–111)
Plant-based diet	Exclusive or predominant consumption of plant-based foods (e.g., vegan or vegetarian diets)	↑*Prevotella, Faecalibacterium, Ruminococcus, Candida*, *Methanobrevibacter*	↑Degradation of complex carbohydrates and SCFA production	A meta-analysis of observational trials found that adherence to vegetarian or vegan diets was linked with lower risk of cardiovascular diseases and cancer	(108–110, 274, 275)
Mediterranean diet	High consumption of fruits, vegetables, and olive oil. Moderate of fish, dairy, and lean meats	↑*Bacteroides, Prevotella, Faecalibacterium*	↑Acetate and propionate	A meta-analysis linked adherence to the Mediterranean diet with a reduction of all-cause mortality	(125, 126)
Fermented foods diet	High consumption of fermented foods (e.g., yogurt, kimchi, cheese, bread)	↑*Lactobacillus* ↑alpha diversity	↑conjugated linoleic acid	Decreased biomarkers of inflammation in a 10-week intervention with a fermented foods diet compared to baseline values	(127, 128)
Low FODMAP diet	Low consumption of rapidly fermentable carbohydrates and polyols	↓*Bifidobacterium* No changes in microbial diversity	No changes in SCFA and BCFA production	Alleviates pain and discomfort in patients suffering from irritable bowel syndrome. May have negative health outcomes	(130, 131)
Low gluten diet	Low consumption of gluten-containing foods	↓*Bifidobacterium*, *Dorea, Veillonellaceae* No changes in microbial diversity	↓Degradation of carbohydrates	Alleviates symptoms in patients with celiac disease or gluten sensitivity. May have negative health outcomes	(132, 133, 276)
Ketogenic diet	High consumption of fat and protein, and restricted consumption of carbohydrates	↓*Bifidobacterium*, *Eubacterium, Faecalibacterium, Roseburia*	↓total SCFAs, acetate, butyrate	Alleviates symptoms in patients with epilepsy and induces fat loss. A systematic review suggested that adherence to Ketogenic diets may increase risk for obesity, type-2 diabetes, and depression	(135)

Observational trials reported that Western diets (rich in fat, protein of animal origin, and simple sugars, and low in complex carbohydrates) were associated with increased fecal abundance of the genera *Alistipes*, *Bacteroides*, and *Penicillium*, and less methanogenic archaea, resulting in higher expression of microbial genes related to bile acid metabolism, amino acid fermentation, and production of BCFAs (108–110). Adherence to this dietary pattern is linked with increased susceptibility to chronic inflammatory, and intestinal, metabolic, neurological, and cardiovascular diseases (111). Consistently, diets high in protein but low in carbohydrates (30% protein, 35% carbohydrate, and 35% fat as calories) and high-fat diets (40% fat as calories) have been linked with perturbations in the colonic microbiota and adverse health outcomes in adults (18, 112, 113). These findings are supported by a systematic review concluding that increased intake of saturated fatty acids, predominantly found in animal-based foods, is associated with reduced colonic microbiota richness and diversity in adults (99). Recently, diets rich in high industrially processed food and low in fiber from plants have been associated with colonic microbiota perturbations, altered profile of plasma metabolites, and an increased risk of disease development, including metabolic disorders and colorectal cancer (19, 114).

On the other hand, diets predominantly based on plants, like vegan and vegetarian diets, are associated with a higher fecal abundance of saccharolytic taxa and enrichment of microbial genes involved in complex carbohydrates degradation and production of SCFAs (108, 110). Notably, colonic microbiota alterations in adults due to adherence to plant-based diets have been linked to protective effects against metabolic and cardiovascular diseases (115). Recently, adherence to a plant-based African heritage diet and consumption of a traditional fermented beverage were associated with lower levels of circulating inflammatory biomarkers in healthy adults (116). These effects may be explained by the high content of non-digestible carbohydrates and polyunsaturated fatty acids in these diets. A meta-analysis concluded that non-digestible carbohydrates support the growth of saccharolytic commensals and stimulate beneficial microbial cross-feeding interactions in adults, producing SCFAs (117). Additionally, the consumption of polyunsaturated fatty acids has been linked with beneficial alterations in colonic microbiota composition in both observational and interventional trials (118, 119).

However, a cross-sectional study found no differences in the fecal concentration of SCFAs and BCFAs between vegans and omnivores (who consumed at least three servings of meat per week) (120). Furthermore, omnivores had higher fecal alpha diversity measured by the Shannon index (120). Consistent with these findings, flexitarian diets (primarily plant-based but including occasional meat, fish, and dairy) have been associated with increased fecal alpha diversity and abundance of beneficial taxa in adults (121–123). These findings suggest that moderate consumption of animal products (e.g., three times per week) alongside a plant-rich diet may offer additional benefits to the colonic microbiota.

In this context, the Mediterranean diet is a prime example of a healthy dietary pattern. This diet is typical of countries around the Mediterranean basin, varying according to the region. It traditionally consists of a high consumption of fruits, vegetables, and olive oil (at least two servings per day), along with a moderate intake of seafood, fish, and dairy (two or more servings per week), and occasional consumption of lean meats (no more than two servings per week) (124). A recent systematic review of observational and interventional trials found that following the Mediterranean diet increased the alpha diversity of the colonic microbiota in adults (125). It also increased the abundance of the genera *Faecalibacterium*, *Prevotella*, and *Bacteroides* and the production of SCFAs, particularly acetate and propionate (125). Additionally, adherence to the Mediterranean diet has been associated with lower mortality rates (126). Similarly, consuming fermented foods, including yogurt, kefir, kimchi, and sourdough bread is encouraged to provide health benefits. Adults consuming diets enriched in fermented foods (at least three servings per week) showed an increased fecal abundance of various *Lactobacillus* species, as well as increased production of conjugated linoleic acid (127). Furthermore, the intake of fermented foods has been associated with increased fecal alpha diversity and lower levels of circulating cytokines (128).

These observations highlight the importance of a balanced and diversified diet to nourish the adult colonic microbiota, aligning with current nutritional recommendations (64). Conversely, restrictions on macronutrients, notably carbohydrates, are associated with perturbations in the colonic microbiota, raising concerns about the potential long-term deleterious effects of such restrictions (129). Restricted diets are typically recommended for managing pre-existing medical conditions. For instance, the low FODMAP diet, characterized by low consumption of rapidly fermentable carbohydrates, is recommended for individuals with disorders of gut-brain interaction (also known as functional gastrointestinal disorders) to alleviate discomfort (130). However, a systematic review and a meta-analysis of interventional studies concluded that this diet reduces the fecal abundance of the genus *Bifidobacterium* in adults with irritable bowel syndrome (131). Similarly, gluten-free or low-gluten diets are recommended for individuals with Celiac Disease or gluten sensitivity. In healthy adults, adherence to these diets has been associated with the decreased fecal abundance of the genera *Bifidobacterium* and *Dorea* and the family *Veillonellaceae*, and decreased expression of microbial genes involved in carbohydrate degradation (132, 133). The ketogenic diet is characterized by high fat intake (70-80% of total energy), moderate protein consumption (10-20% of total energy), and restriction of carbohydrates (less than 10% of total energy). Variants of this diet are recommended for individuals with epilepsy (for example, the medium-chain triglyceride diet) or following a rapid weight-loss strategy (134). A systematic review concluded that this diet decreases the fecal abundance of the *Bifidobacterium* genus and potentially reduces the abundance of butyrate-producing genera, leading to reduced levels of acetate and butyrate (135). These alterations in the colonic microbiota may contribute to an increased risk of obesity, type-2 diabetes, and depression (135). Further evidence supports these adverse outcomes, as adherence to the ketogenic diet reduced glucose tolerance in healthy adults (136).

### 4.3 Older adulthood

The colonic microbiota remains stable during adulthood until it undergoes modifications in its composition and function at around 65 years old, when changes in lifestyle and physiology occur with aging. The metabolism and physical activity levels decrease, antibiotics are used more frequently, the diet becomes less diversified, colon motility decreases, and fecal retention time increases (137). Observational evidence suggests that older adults have high inter-individual variation in the colonic microbiota, which is strongly influenced by their health status (138).

Healthy older adults have fecal microbiota similar to that of healthy young adults, although observational studies suggest increased diversity of methanogenic archaea and lower viral richness (139–141). On the other hand, unhealthy aging (e.g., frailty or chronic diseases) is associated with a reduced abundance of the genera *Bifidobacterium*, *Faecalibacterium*, and *Eubacterium*, and an increased abundance of the family *Enterobacteriaceae* and genera *Streptococcus, Clostridium, Penicillium, Candida*, and *Aspergillus* (142–144). In addition, the expression of genes synthesizing vitamins and fatty acids is reduced (143). These alterations in the colonic microbiota are associated with chronic inflammation and an increased risk of morbidity and mortality (145).

A recent systematic review evaluated the effect of different dietary patterns on the fecal microbiota of older adults, including a total of 38 intervention trials, most of which profiled the microbiota using 16S rRNA sequencing (146). Diets rich in plant foods and including animal products in moderation (such as daily consumption of dairy and lean meats or fish two to four times per week) increased the fecal abundance of saccharolytic taxa and the production of SCFAs (146). Similarly, an observational study that evaluated the fecal microbiota of older adults using shotgun metagenomic sequencing found that adherence to the healthy plant-based diet index was associated with a greater fecal abundance of bacterial saccharolytic species, along with enriched pathways for the biosynthesis of branched-chain amino acids (147). These results may be explained by the high content of non-digestible carbohydrates and phytochemicals in these diets. For instance, greater consumption of dietary fiber among older adults was associated with an increased fecal abundance of the order Clostridiales, which includes butyrate-producing bacteria, and increased expression of pathways involved in polysaccharide degradation, according to an observational study employing shotgun metagenomic sequencing (148). Furthermore, an intervention trial reported that adherence to a polyphenol-rich diet (total polyphenols around 1,300 mg/day) increased the fecal abundance of butyrate-producing bacteria, as profiled by 16S rRNA sequencing, and reduced blood pressure in older adults (149).

In contrast, the systematic review of intervention trials found that high intakes of fat, protein, and simple sugars but low consumptions of complex carbohydrates are associated with the growth of opportunistic pathogens, production of pro-inflammatory toxins, and frailty (146). Furthermore, an intervention study that profiled the fecal microbiota of older adults using 16S rRNA sequencing found that diets rich in fat but low in carbohydrates have been associated with decreased abundance of the genus *Bifidobacterium* (150). Consistently, a longitudinal study in older adults linked higher red meat intake to increased plasma concentration of trimethylamine N-oxide, a microbial metabolite associated with cardiovascular risk (151). These observations are consistent with results observed for young adults, highlighting the importance of a diversified diet primarily composed of fruits, vegetables, and whole cereals to nourish beneficial colonic microbes (Table 3). In line with these findings, a longitudinal study of over 100,000 participants followed for 30 years assessed the impact of long-term dietary patterns on aging. Diets rich in plant-based foods, such as fruits, vegetables, whole grains, and nuts, and including animal-based foods in moderation, like low-fat dairy, were associated with healthy aging, defined as survival to the age of 70 years with intact cognitive, physical, and mental functions, and without chronic diseases (152).

**TABLE 3:** Impact of various dietary patterns on the colonic microbiota of older adults.

Diet	Definition	Microbial composition	Microbial function	Observed effect of diet on host health	Reference
Western diet	High consumption of processed meats and refined grains	↑*Alistipes, Desulfovibrio, Ruminococcus* ↓*Faecalibacterium, Prevotella*	↑Amino acid metabolism ↓SCFA production	Adherence to a Western diet was associated with increased body mass index compared to a Prudent diet	(17)
High-protein diet	Consumption of protein higher than the recommended dietary intake (> 0.8 g protein/kg bodyweight/day)	No changes in taxa abundance or microbial diversity	No changes in the production of organic acids	No changes in appetite after 6-moth intervention compared to control (habitual diet)	(158, 159)
Prudent diet	High consumption of fruits, vegetables, nuts, fish, and chicken	↑*Clostridium, Faecalibacterium, Lachnospira* ↓*Desulfovibrio, Ruminococcus*	↑Complex carbohydrate degradation and SCFA production	Adherence to a Prudent diet was associated with reduced body mass index compared to a Western diet	(17)
Mediterranean diet	High consumption of fruits, vegetables, and olive oil. Moderate of fish, dairy, and lean meats	↑*Roseburia, Eubacterium, Faecalibacterium* ↓*Ruminococcus torques*	↑SCFA and BCFA production ↓Secondary bile acids, p-cresol, ethanol	Reduced frailty and chronic inflammation, and improved cognitive function after 1-year intervention compared to control (habitual diet)	(277)
Polyphenol-rich diet	High consumption of polyphenol-rich foods (e.g., berries, pomegranate, green tea, dark chocolate)	↑*Faecalibacterium, Butyricicoccus, Ruminococcaceae* ↓*Streptococcus*, *Enterobacteriaceae*	Not evaluated	Increased serum concentration of indole 3-propionic acid and decreased of zonulin after 8-week intervention compared to the control diet (low-polyphenol diet)	(149, 278)

However, increased protein consumption appears to be beneficial in older adults, who typically have slower protein digestion and absorption compared to younger individuals (153). For instance, a cross-sectional analysis of the fecal microbiota of older men, characterized using 16S rRNA gene sequencing, revealed that higher protein intake was associated with increased fecal alpha diversity (154). Additionally, a meta-analysis of observational trials concluded that protein intake was negatively associated with frailty in older individuals (155). Therefore, a daily intake of 1.2 g protein/kg bodyweight, compared to the standard recommendations of 0.66-0.8, has been proposed to support good health and maintain functionality in older populations (156). In line with this recommendation, a narrative review suggested that high-fiber diets enriched with protein from legumes, dairy, and lean meats could promote a balanced colonic microbiota (eubiosis), also contributing to muscle synthesis and overall metabolic health in older adults (157). In this context, complex carbohydrates are important to mitigate excessive protein fermentation in the colon and the consequent production of deleterious metabolites, such as hydrogen sulfide and nitrogen-derivatives (92, 93). As demonstrated by intervention trials, protein intakes exceeding the recommended dietary allowance did not alter fecal microbiota composition, characterized using 16S rRNA sequencing, or function in older adults when accompanied by a prudent diet (158, 159).

### 4.4 Centenarian age

Recently, colonic microbiota investigations have focused on centenarians (individuals aged 100 or older) as models for healthy aging. Compared to younger controls, observational studies demonstrated that centenarians exhibit higher fecal bacterial and viral diversity (160–162). A systematic review of 27 observational studies suggests that their high fecal microbial diversity and abundance of health-promoting taxa contribute to healthy aging and longevity (163).

In terms of microbial composition, the genera *Alistipes*, *Parabacteroides*, *Clostridium*, and *Methanobrevibacter* are enriched in the feces of healthy centenarians, while the butyrate-producing species *Faecalibacterium prausnitzii* and *Eubacterium rectale* are depleted (160, 162, 164). However, no age-related changes have been observed in the fecal fungal microbiota (165). Concerning functionality, centenarians have lower fecal butyrate concentrations but higher levels of BCFAs, ammonium, and secondary bile acids compared to younger controls (160). Furthermore, their fecal microbiome is enriched in genes associated with SCFA production from amino acids, secondary bile acid metabolism, and the degradation of xenobiotics, plant-based fats, and tryptophan (166–168). In contrast, they have fewer genes involved in carbohydrate and animal fat metabolism (166–168).

Few investigations have assessed the interaction between diet and colonic microbes in centenarians, with current knowledge primarily derived from longitudinal and cross-over studies. Overall, adherence to a diverse, plant-rich diet has been associated with a higher abundance of microbial taxa linked to longevity, as observed in Italian, Chinese, and South Korean centenarians (166, 169–172). Additionally, the consumption of fermented soybean paste was positively associated with the distinct fecal microbiota composition of South Korean centenarians, according to an observational study that profiled the microbiota using 16S rRNA gene sequencing (172). A cross-sectional study of Estonian centenarians found that cereal consumption and lower adherence to Western dietary patterns were linked to longevity (162). These observations highlight the importance of a prudent, plant-rich diet for healthy aging, aligning with dietary patterns that support a balanced colonic microbiota across life stages.

Interestingly, Estonian centenarians were also more exposed to animals and experienced lower sanitary conditions during their childhood (162). Similarly, early-life exposure to animals has been shown to contribute to the development of the fecal microbiota in infancy (25). Taken together, these findings suggest that early exposure to environmental microbes may play a crucial role in supporting the long-term balance of colonic microbiota throughout life.

## 5 Dietary recommendations for nourishing a balanced colonic microbiota

**FIGURE 2:**
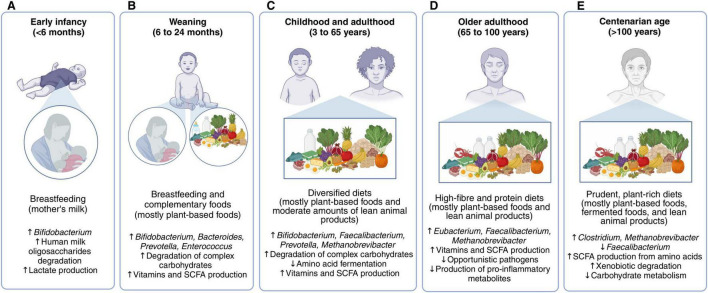
Favorable diets for nourishing the colonic microbiota in different life stages. In early infancy **(A)**, breastfeeding is the optimal dietary strategy, but mother’s milk does not meet all the necessary nutritional requirements once the infant reaches the weaning period. In this stage **(B)**, diets should combine breastfeeding with complementary foods, mainly fruits, vegetables, whole cereals, and animal products in moderation. The colonic microbiota matures as the introduction of solid foods increases and breastmilk or infant-formula decreases, achieving an adult state in which breast milk is no longer consumed. Children, adolescents, and adults have similar diets for promoting a balanced microbiota **(C)**. These are rich in non-digestible carbohydrates, phytochemicals, and polyunsaturated fatty acids found in fruits, vegetables, nuts, pulses and whole grains, also containing moderate amounts of fish, fermented dairy products, and lean meats. A higher intake of protein is recommended for older adults, particularly from dairy and lean meats **(D)**. Centenarians have a similar dietary pattern to that of older adults **(E)**.

The evidence gathered in this review suggests that diets promoting a balanced colonic microbiota in infants post-weaning, adults, older adults, and centenarians share similar compositions (Figure 2). These diets are diverse and primarily based on plant foods, such as fruits, vegetables, and whole grains. These foods are rich in complex carbohydrates, polyunsaturated fatty acids, and polyphenols, which support the growth of saccharolytic microbes, for example, taxa from the genera *Bifidobacterium*, *Faecalibacterium*, *Prevotella*, *Eubacterium*, *and Ruminococcus*, and the production of beneficial metabolites, such as SCFAs and conjugated fatty acids (76, 117, 119). Additionally, polyunsaturated fatty acids and polyphenols have anti-inflammatory and antioxidant properties, and their metabolism by colonic microbes may confer further benefits to the host (76, 173). For instance, a 2-month intervention in adults consuming cereals enriched with polyphenols, dietary fiber, and omega-3 fatty acids increased the fecal abundance of *Bacteroides* species while reducing fecal calprotectin levels, a biomarker for intestinal inflammation (174).

In addition to plant-based foods, favorable diets also include moderate consumption of animal-based foods, such as fish and lean meats (two to four servings per week). These foods are rich sources of essential amino acids and micronutrients, supporting colonic microbial diversity and contributing to meeting nutritional needs (120). Their consumption is particularly important for infants and older adults to promote physiological development and reduce disease risk (155). Daily consumption of fermented foods, such as fermented dairy, is also encouraged, as they contain lactic acid bacteria, supporting colonic eubiosis (127). Importantly, the intake of animal foods should be paired with the consumption of complex carbohydrates to mitigate excessive microbial fermentation of animal protein and fat. Notably, these recommendations for nourishing the colonic microbiota align with current dietary guidelines (64).

## 6 Limitations and future perspectives in diet-colonic microbiota research

Most of the knowledge on diet-colonic microbiota interaction in humans comes from studies using fecal samples as proxies. Compared to biopsies, fecal sampling is a non-invasive and cost-effective approach (175). However, they predominantly represent microbial communities from the distal colon, providing limited information about microbes attached to the colon’s mucosa or inhabiting other parts of the gastrointestinal tract (176). As a result, diet-induced changes observed in fecal microbial function and composition may not accurately reflect microbial alterations throughout the entire large intestine. Furthermore, current culture and sequencing techniques are unable to fully characterize the human colonic microbiota (177). Ultimately, due to technical limitations, our current understanding of how dietary patterns influence colonic commensals remains largely inferred and may not fully capture the complexity of host-microbe interactions.

Diet-colonic microbiota research has traditionally focused on adults. Hence, there is an opportunity for further research to expand our knowledge on diet-microbiota interactions in other life stages, such as infancy and older adulthood. Notably, weaning plays a critical role in colonic microbiota development (72). Investigating the impact of complementary feeding on host-microbiota associations early in life is a promising field to promote health and wellbeing and prevent diseases. Similarly, the continuous rise in human life expectancy urges more comprehension of the relationship between colonic microbiota and aging. The confounding effects of diseases on colonic microbes often challenge research on older populations. Therefore, studies involving both healthy and unhealthy older adults are necessary to deepen our understanding of how diet-microbiota modulations can support functionality during aging.

Among dietary compounds, the effects of complex carbohydrates on colonic microbes are well-characterized, whereas less is known about protein, fatty acids, polyphenols, micronutrients, and food additives. Notably, polyphenols and polyunsaturated fatty acids have been associated with health benefits, in which colonic microbes seem to play a critical role (76, 119). A better understanding of the impact of these nutrients on the colonic microbiota is needed, including in the long term. Future research should also investigate how dietary compounds interact with each other and their combined effects on the microbiota. Increased knowledge of the role of various nutrients in shaping colonic microbes, alongside individual factors (e.g., age, health status, activity level), will help researchers and medical professionals to tailor nutritional recommendations based on individual needs.

These challenges highlight the need for complementary methods to study the influence of dietary patterns on the colonic microbiota. Randomized clinical trials are the gold standard approach to measure the effect of food interventions on colonic microbes and resulting host health outcomes. They are also useful for validating findings from animal models and *in vitro* studies. However, clinical trials are time and resource-consuming, are prone to confounding factors, and rely on participant compliance and the accuracy of food questionnaires, limiting their feasibility (178). Furthermore, dietary assessment tools vary across microbiome studies, highlighting the need for standardized methods to improve comparability between studies targeting different age groups.

A promising strategy to address these limitations is to combine traditional methodologies with mathematical modeling. Mathematical models can investigate hypotheses that cannot be efficiently evaluated *in vitro* or *in vivo*, using a fraction of the time and cost of traditional approaches. *In silico* pipelines for predicting the effect of diets on personalized microbial communities have already been proposed (179, 180). Ongoing development and validation of these models could expand colonic microbiota research to traditionally underrepresented populations. For instance, *in silico* approaches were used to predict compositional and functional changes in the colonic microbiota of infants, children with different clinical conditions, healthy adults, and adults with Crohn’s disease according to the diet (89, 181–184). Moreover, models are flexible and can create personalized simulations based on input data, contributing toward personalized nutrition. Ultimately, integrating mathematical models with traditional approaches can reduce the costs of colonic microbiota research and accelerate our understanding of the relationship between diet, colonic microbes, and host health.

## 7 Conclusion

Colonic microbes ferment non-absorbed dietary compounds producing bioactive metabolites that influence host physiology. Therefore, identifying dietary patterns that support colonic eubiosis across different life stages is crucial for promoting host health and well-being. The evidence gathered in this review suggests that diets nourishing the colonic microbiota throughout human life are primarily composed of plant-based foods and include daily consumption of fermented foods, such as dairy products, and moderate amounts of fish and lean meats (two to four times a week). However, most diet-colonic microbiota investigations have focused on adults, neglecting weaning infants and older adults. Notably, weaning is a critical period for colonic microbiota development, setting the foundation for later life. In older adulthood, colonic microbes have a crucial role in maintaining functionality and promoting healthy aging. The limited understanding of how diets influence colonic microbes of infants and older adults is a significant barrier to using colonic microbiota modulation strategies to promote health. Further investigation of the long-term effects of dietary patterns on colonic microbes across different life stages is necessary to overcome some of the current limitations in diet-colonic microbiota research.
