# Supplementary material for: Dietary patterns influencing the human colonic microbiota from infancy to centenarian age: a narrative review
Source: Front Nutr. 2025 Jun 4;12:1591341. doi: 10.3389/fnut.2025.1591341 (PMC12174164; doi:10.3389/fnut.2025.1591341)
Supplement: Supplementary file 1 [file Data_Sheet_1.pdf]

Supplementary Material

Supplementary Table 1. Principal bacteria, archaea, and fungi composing the human colonic microbiota

Phylum	Genus	Characteristics	Colonic abundance and observed effect on host health			Reference
			Infants	Adults	Older adults	
Bacillota	*Streptococcus*	Saccharolytic and proteolytic, produces lactate and acetate. Includes both beneficial species (e.g. *S. thermophilus*) and pathogens (e.g. *S. bovis*)	Infants delivered by C-section and formula-fed had an increased abundance of *Streptococcus* compared to other infants (n=27)	*Streptococcus* abundance was positively associated with coronary atherosclerosis (n=8973)	A higher abundance of *Streptococcus* was associated with unhealthy aging (n=32)	(190–192)
	Lactobacillus	Mainly saccharolytic, produces lactate and bacteriocins. Species are typically considered beneficial (e.g. *L. rhamnosus*)	Vaginally delivered infants had a higher abundance of *Lactobacillus* compared to those delivered via C-section during the first days of life (n=37)	A higher abundance of *Lactobacillus* was associated with type-2 diabetes (n=18) and obesity (n=20) in adults	Long-living older adults (aged 97-100) had increased *Lactobacillus* abundance compared to healthy older adults (aged 60-76) (n=20)	(193–196)
	Clostridium	Saccharolytic and proteolytic, produces acetate, propionate, and butyrate. Contains both beneficial species (e.g. *C. butyricum*) and pathogens (e.g. *C. difficile*)	Higher abundance of *Clostridium sensu stricto* in infants with food allergies (n=34)	A higher abundance of *Clostridium* was associated with obesity in adults (n=307)	Older adults with Parkinson's disease had a decreased abundance of *Clostridium* compared to healthy controls (n=45)	(197–199)
	Ruminococcus	Mainly saccharolytic, produces acetate. Degrades resistant starch contributing to butyrate production via cross-feeding (e.g. *R. bromii*)	Lower abundance of *Ruminococcus* in children and adolescents with Crohn's disease (n=64)	Lower abundance of *Ruminococcus* in adults with Crohn's disease compared to healthy controls (n=10)	Higher abundance of *Ruminococcus* in older adults with frailty and sarcopenia (n=18)	(200–202)
	Faecalibacterium	Mainly saccharolytic, produces SCFA, including butyrate. Typically considered beneficial, some species produce anti-inflammatory molecules (e.g. *F. prausnitzii*)	Lower abundance of *F. prausnitzii* in children with allergic asthma (n=92)	Lower abundance of *F. prausnitzii* in adults with Crohn's disease (n=68)	Lower abundance of *F. prausnitzii* in older adults with mild-cognitive impairment (n=15)	(203–205)
	Enterococcus	Saccharolytic and proteolytic, produces lactate and acetate. Some species raise concern due to capacity to acquire antibiotic resistance (e.g. *E. faecalis*)	Higher abundance of *Enterococcus* in infants with food allergies (n=34)	Higher abundance of *E. faecalis* in adults with colorectal cancer (n=25)	Higher abundance of *Enterococcus* in older adults with Parkinson's disease (n=24)	(199,206,207)
	Eubacterium	Chemoheterotroph, produces SCFAs, including butyrate.	Lower abundance of *E. rectale* in children with	Lower abundance of *Eubacterium* in adults with	Lower abundance of *Eubacterium* in older	(201,202,208,209)

		Typically associated with the promotion of colonic homeostasis (e.g. *E. rectale*)	ulcerative colitis (n=6) and in children with neurodevelopmental disorders (n=36)	Crohn's disease compared to healthy controls (n=10)	adults with frailty and sarcopenia (n=18)	
Bacteroidota	*Bacteroides*	Saccharolytic and proteolytic, produces acetate, propionate, and succinate. Contains both beneficial species (e.g. *B. thetaiotaomicron*) and pathogens (e.g. *B. fragilis*)	Decreased abundance of *Bacteroides* in infants delivered via C-section compared to infants vaginally delivered (n=9)	A meta-analysis reported lower abundance of *Bacteroides* in adults and adolescents with Crohn's disease and ulcerative colitis (n=706)	Higher abundance of *Bacteroides* in older adults was positively associated with increased risk of all-cause mortality (n=706)	(138,210,211)
	Prevotella	Saccharolytic and proteolytic, produces acetate and propionate. Contains species with potential role in promoting (e.g. *P. copri*) or suppressing inflammation (e.g. *P. histicola*)	Higher abundance of *Prevotella* in infants with food allergies (n=34)	Higher abundance of *Prevotella* in adults with hypertension (n=99). Increased abundance of *Prevotella* was associated with improved glucose metabolism in healthy adults (n=10)	A systematic review reported a lower abundance of *Prevotella* in frail older adults (n=912)	(199,212–214)
	Alistipes	Saccharolytic and proteolytic, produces acetate and propionate. Contains pathogenic species that produce pro-inflammatory toxins (e.g. *A. finegoldii*)	Lower abundance of *A. putredinis* in infancy was associated with neurodevelopmental disorders later in life (n=1748)	Lower abundance of *Alistipes* in adults with atrial fibrillation (n=50) and higher abundance in adults with chronic fatigue syndrome (n=25)	Higher abundance of *Alistipes* in older adults with frailty compared to healthy controls (n=47)	(215–218)
Actinomycetota	*Bifidobacterium*	Saccharolytic, produces acetate and lactate. Predominant in the infant colon. Typically considered beneficial (e.g. *B. breve* and *B. longum*)	Vaginally delivered and breastfed infants have higher *Bifidobacterium* abundance compared to those delivered via C-section and formula-fed (n=8)	Lower abundance of *Bifidobacterium* in obese women (n=15)	Higher abundance of *Bifidobacterium* in older adults with frailty compared to healthy controls (n=47)	(192,217,219)
Pseudomonadota	*Escherichia*	Saccharolytic and proteolytic. Contains pathogenic species that produce pro-inflammatory toxins (e.g. *E. coli*)	Higher abundance of *Escherichia* in children with non-alcoholic steatohepatitis (n=22)	A meta-analysis reported higher abundance of *E. coli* in adults with irritable bowel syndrome compared to healthy controls (n=1340)	Higher abundance of *Escherichia-Shigella* in critically ill older adults (n=72)	(220–222)
	Desulfovibrio	Reduces sulphate, producing hydrogen sulphide (excessive production is deleterious)	Higher abundance of *Desulfovibrio* in infants with stunting compared to healthy controls (n=10)	Higher abundance of *Desulfovibrio* in adults with systemic sclerosis (n=59)	Higher abundance of *Desulfovibrio* in older adults with Parkinson's disease (n=20)	(223–225)
Verrucomicrobiota	*Akkermansia*	Mucin degrader. Produces acetate, propionate, and butyrate. Assumed to promote colonic barrier integrity (e.g. *A. muciniphila*)	Lower abundance of *A. muciniphila* in overweight children (n=20)	Lower abundance of *A. muciniphila* in pregnant women (n=16) and patients with inflammatory bowel disease (n=46)	Higher abundance of *Akkermansia* in older adults with frailty compared to healthy controls (n=47)	(217,226–228)
Euryarchaeota	*Methanobrevibacter*	Archaea. Consumes hydrogen to produce methane, facilitating fermentation by anaerobic	Lower abundance of *Methanobrevibacter* in children with severe acute malnutrition (n=143)	Higher abundance of *Methanobrevibacter* in anorexic adults (n=20)	Abundance of *M. smithii* in older adults was positively associated with the severity of	(193,229,230)

		saccharolytic bacteria (e.g. *M. smithii*)			cognitive impairment (n=159)	
Ascomycota	*Candida*	Fungi. Converts simple carbohydrates into ethanol and acetate. Contains opportunistic pathogens (e.g. *C. albicans*)	Higher abundance of *Candida* in children with autism compared to neurotypical controls (n=40)	Higher abundance of *Candida* in adults with inflammatory bowel disease (n=235)	Higher abundance of *C. tropicalis* in older adults with Alzheimer's disease (n=88)	(231–233)
	Saccharomyces	Fungi. Converts simple carbohydrates into ethanol and carbon dioxide. Typically considered commensals (e.g. *S. cerevisiae*)	Higher abundance of *S. cerevisiae* in children with autism compared to neurotypical controls (n=29)	Higher abundance of *Saccharomyces* in adults with colorectal cancer (n=71). Lower abundance of *Saccharomyces* in adults with inflammatory bowel disease (n=235)	*Saccharomyces* abundance was positively associated with higher levels of circulating plasma triglycerides and very low-density lipoprotein in older adults (n=99)	(142,231,234,235)

Supplementary Table 2. Principal metabolites produced by the human colonic microbiota

Category	Compound	Substrate or precursor	Major producing microbial taxa	Observed effect on host health			Reference
				Infants	Adults	Older adults	
Short-chain fatty acids (SCFAs)	Acetate	Primarily dietary fibre and resistant starch, but also amino acids	*Bifidobacterium, Lactobacillus, Prevotella, Ruminococcus, A. muciniphila*	Infants exclusively breastfed had a lower concentration of acetate, propionate, and butyrate (n=48)	A meta-analysis found an increased fecal concentration of acetate, propionate, and butyrate in obese adults (n=221)	Lower concentration of fecal acetate, propionate, and butyrate in older adults with Alzheimer's disease compared to healthy controls (n=27)	(236–238)
	Propionate		*Prevotella, Bacteroides, Propionibacterium*				
	Butyrate		*Clostridium, Roseburia, F. prausnitzii, E. rectale, B. fragilis, R. bromii, A. muciniphila*				
Branched-chain fatty acids (BCFAs)	Isobutyrate and isovalerate	Amino acids (valine, leucine, and isoleucine)	*Bacteroides, Clostridium*	Increased fecal levels of isobutyrate and isovalerate in formula-fed infants compared to breastfed infants (n=33)	Increased isobutyrate fecal levels in adults with non-alcoholic fatty liver disease (n=24) and increased isovalerate in adults with depression (n=34)	Increased fecal levels of isobutyrate and isovalerate in older adults with colorectal cancer (n=50)	(239–242)
Conjugated fatty acids	Conjugated linoleic acid	Linoleic acid	*Bifidobacterium, Enterobacter, Lactobacillus, Clostridium*	Conjugated linoleic acid supplementation in obese children decreased body fat and high-density lipoprotein compared to placebo (n=28)	Conjugated linoleic acid supplementation in adults decreased T lymphocyte activation (n=39)	Conjugated linoleic acid supplementation in adults older with type 2 diabetes reduced insulin sensitivity (n=16)	(243–245)
Vitamins	Complex B vitamins	Carbohydrates and amino acids	*Bifidobacterium, Lactobacillus, Bacteroides*	Deficiency of complex B vitamins in infants was linked with compromised brain development (n=6)	Decreased serum levels of vitamin B7 in adults were associated with obesity and type 2 diabetes (n=24)	Deficiency of complex B vitamins in older adults was linked with increased risk of dementia (n=228)	(246–248)
	Vitamin K family		*Bacteroides, Prevotella*	Vitamin K family deficiency in infants was linked with convulsions, hemorrhage, and death (n=30)	Serum vitamin K_1 levels were negatively associated with circulating inflammatory biomarkers in adults (n=1381)	Serum vitamin K_1 levels were negatively associated with circulating inflammatory biomarkers in older adults (n=662)	(249–251)
Gases	H_2	Carbohydrates	*Clostridium, Enterobacteriaceae*	Excessive H_2 production has been associated with the development of colic symptoms in infants (n=8)	Higher H_2 production from *in vitro* starch fermentation using fecal inoculum from adults with irritable bowel syndrome compared to healthy controls (n=14)	No differences in H_2 production from *in vitro* fermentation of different carbohydrates using fecal inoculum from older adults with pre-frailty compared to young controls (n=6)	(252–254)
	CO_2		*Clostridium*	No changes in CO_2 production were observed during *in vitro* incubation of fecal inoculum from infants fed soy-based infant formula,	CO_2 insufflation during colonoscopy in adults reduced the fecal abundance of colonic	CO_2 insufflation during colonoscopy in older adults was associated with less pain compared to air insufflation (n=66)	(255–257)

				milk-based formula, or breastmilk (n=18)	pathogens compared to air insufflation (n=38)		
	CH_4	H_2, CO_2	*Methanobrevibacter smithii*	Higher breath methane in children with chronic constipation compared to healthy controls (n=75)	Higher breath methane in adults with multiple sclerosis compared to healthy controls (n=60)	Higher CH_4 production from *in vitro* fermentation of different carbohydrates using fecal inoculum from older adults with pre-frailty compared to young controls (n=6)	(254,258,259)
	H_2S	Sulphate	*Desulfovibrio*	Higher H_2S production was observed from *in vitro* incubation of fecal inoculum from infants fed soy-based infant formula compared to breastfed infants (n=5)	Higher H_2S production from *in vitro* starch fermentation using fecal inoculum from adults with irritable bowel syndrome compared to healthy controls (n=14)	A meta-analysis reported lower levels of circulating H_2S in older adults with chronic and degenerative diseases compared to healthy controls (n=1721)	(253,255,260)
Secondary bile acids	Deoxycholic acid, lithocholic acid	Primary bile acids	*Clostridium, Bifidobacterium, Lactobacillus, Bacteroides, M. smithii*	Higher fecal levels of secondary bile acid in critically ill children compared to healthy controls (n=39)	Higher levels of circulating lithocholic acid in adults with severe obstructive coronary heart disease compared to angiographically normal controls (n=150)	Higher levels of circulating unconjugated secondary bile acids were associated with a higher risk for cardiovascular diseases in older adults with type 2 diabetes (n=1234)	(261,262)
	Ursodeoxycholic acid	Primary bile acids	*Ruminococcus, Clostridium*	Higher levels of circulating ursodeoxycholate in formula-fed infants compared to breastfed infants (n=48)	Ursodeoxycholic acid use was associated with a reduced risk of colorectal cancer in adults (n=2557)	Ursodeoxycholic acid use was associated with a reduced risk of colorectal cancer in older adults (n=1911)	(263–265)
Neurotransmitters	Dopamine	Tyrosine, 3,4-dihydroxy-L-phenylalanine	*Bacillus, E. coli, Staphylococcus*	Higher circulating serotonin and serotonin transporter levels in children with autism compared to healthy control (n=60)	Higher plasma levels of dopamine and gamma-aminobutyric acid in adults with major depressive disorder compared to healthy controls (n=49)	Alterations in the dopamine system were associated with the progression of Alzheimer's disease in older adults (n= 144)	(266–268)
	Norepinephrine	Tyrosine	*Bacillus*				
	Serotonin	Tryptophan, 5-hydroxytryptophan	*Lactobacillus, Streptococcus, Clostridium*				
	Gamma-aminobutyric acid	Acetate, glutamate	*Bifidobacterium, Lactobacillus, Eubacterium, Bacteroides*				
Nitrogen-derivatives	Ammonia	Amino acids and peptides	*Clostridium, Fusobacterium, Bacteroides*	Hyperammonemia in infants was associated with liver failure and urea cycle defects (n=90)	Higher circulating levels of ammonia were associated with hepatic steatosis in adults (n=25)	Higher blood ammonia levels in patients with Alzheimer's disease compared to controls (n=3)	(269–271)
	p-cresol	Tyrosine	*Fusobacterium, Enterobacter, Clostridium*	Higher urinary levels of p-cresol in children with autism compared to healthy controls (n=33)	Higher circulating levels of p-cresol in adults undergoing hemodialysis were associated with increased risk for infection-	Higher circulating levels of p-cresol in older adults undergoing hemodialysis compared to non-hemodialysis controls (n=4)	(272–274)

					related hospitalizations (n=464)		
	Indole	Tryptophan	*Peptostreptococcus, Akkermansia, Clostridium*	Fecal levels of indole-3-lactic acid correlated positively with increased fecal abundance of *Bifidobacterium infants* in breastfed infants (n=18)	Lower serum levels of indole-3-pyruvic acid in adults with ulcerative colitis compared to healthy controls (n=15)	Lower fecal levels of indole-3-pyruvic acid in older adults with Alzheimer's disease compared to healthy controls (n=27)	(238,275,276)
Endotoxins	Lipopolysaccharide	Lipid A, oligosaccharide, O antigen	*Enterobacteriaceae, Bacteroidales*	Increased exposure to lipopolysaccharides in early infancy was associated with the development of autoimmune diseases (n=168)	Lipopolysaccharide exposure increased intestinal permeability in healthy adults (n=14)	Trend towards higher circulating levels of lipopolysaccharides in older adults with Alzheimer's disease compared to healthy controls (n=27)	(238,277,278)

References

138. Wilmanski T, Diener C, Rappaport N, Patwardhan S, Wiedrick J, Lapidus J, Earls JC, Zimmer A, Glusman G, Robinson M, et al. Gut microbiome pattern reflects healthy ageing and predicts survival in humans. *Nat Metab* (2021) 3:274–286. doi: 10.1038/s42255-021-00348-0

142. Ahmad HF, Mejia JLC, Krych L, Khakimov B, Kot W, Bechshøft RL, Reitelseder S, Højfeldt GW, Engelsen SB, Holm L, et al. Gut Mycobiome Dysbiosis Is Linked to Hypertriglyceridemia among Home Dwelling Elderly Danes. (2020)2020.04.16.044693. doi: 10.1101/2020.04.16.044693

190. Singh H, Torralba MG, Moncera KJ, DiLello L, Petrini J, Nelson KE, Pieper R. Gastro-intestinal and oral microbiome signatures associated with healthy aging. *GeroScience* (2019) 41:907–921. doi: 10.1007/s11357-019-00098-8

191. Sayols-Baixeras S, Dekkers KF, Baldanzi G, Jönsson D, Hammar U, Lin Y-T, Ahmad S, Nguyen D, Varotsis G, Pita S, et al. Streptococcus Species Abundance in the Gut Is Linked to Subclinical Coronary Atherosclerosis in 8973 Participants From the SCAPIS Cohort. *Circulation* (2023) 148:459–472. doi: 10.1161/CIRCULATIONAHA.123.063914

192. Ma J, Li Z, Zhang W, Zhang C, Zhang Y, Mei H, Zhuo N, Wang H, Wu D. Comparison of the Gut Microbiota in Healthy Infants With Different Delivery Modes and Feeding Types: A Cohort Study. *Front Microbiol* (2022) 13: doi: 10.3389/fmicb.2022.868227

193. Armougom F, Henry M, Vialettes B, Raccah D, Raoult D. Monitoring Bacterial Community of Human Gut Microbiota Reveals an Increase in Lactobacillus in Obese Patients and Methanogens in Anorexic Patients. *PLOS ONE* (2009) 4:e7125. doi: 10.1371/journal.pone.0007125

194. Larsen N, Vogensen FK, Berg FWJ van den, Nielsen DS, Andreasen AS, Pedersen BK, Al-Soud WA, Sørensen SJ, Hansen LH, Jakobsen M. Gut Microbiota in Human Adults with Type 2 Diabetes Differs from Non-Diabetic Adults. *PLOS ONE* (2010) 5:e9085. doi: 10.1371/journal.pone.0009085

195. Mitsou EK, Kirtzalidou E, Oikonomou I, Liosis G, Kyriacou A. Fecal microflora of Greek healthy neonates. *Anaerobe* (2008) 14:94–101. doi: 10.1016/j.anaerobe.2007.11.002

196. Kashtanova DA, Klimenko NS, Strazhesko ID, Starikova EV, Glushchenko OE, Gudkov DA, Tkacheva ON. A Cross-Sectional Study of the Gut Microbiota Composition in Moscow Long-Livers. *Microorganisms* (2020) 8:1162. doi: 10.3390/microorganisms8081162

197. Zeng Q, Li D, He Y, Li Y, Yang Z, Zhao X, Liu Y, Wang Y, Sun J, Feng X, et al. Discrepant gut microbiota markers for the classification of obesity-related metabolic abnormalities. *Sci Rep* (2019) 9:13424. doi: 10.1038/s41598-019-49462-w

198. Hasegawa S, Goto S, Tsuji H, Okuno T, Asahara T, Nomoto K, Shibata A, Fujisawa Y, Minato T, Okamoto A, et al. Intestinal Dysbiosis and Lowered Serum Lipopolysaccharide-Binding

Protein in Parkinson’s Disease. *PLOS ONE* (2015) 10:e0142164. doi: 10.1371/journal.pone.0142164

199. Ling Z, Li Z, Liu X, Cheng Y, Luo Y, Tong X, Yuan L, Wang Y, Sun J, Li L, et al. Altered Fecal Microbiota Composition Associated with Food Allergy in Infants. *Appl Environ Microbiol* (2014) 80:2546–2554. doi: 10.1128/AEM.00003-14

200. Kowalska-Duplaga K, Gosiewski T, Kapusta P, Sroka-Oleksiak A, Wędrychowicz A, Pieczarkowski S, Ludwig-Słomczyńska AH, Wołkow PP, Fyderek K. Differences in the intestinal microbiome of healthy children and patients with newly diagnosed Crohn’s disease. *Sci Rep* (2019) 9:18880. doi: 10.1038/s41598-019-55290-9

201. Takahashi K, Nishida A, Fujimoto T, Fujii M, Shioya M, Imaeda H, Inatomi O, Bamba S, Andoh A, Sugimoto M. Reduced Abundance of Butyrate-Producing Bacteria Species in the Fecal Microbial Community in Crohn’s Disease. *Digestion* (2016) 93:59–65. doi: 10.1159/000441768

202. Picca A, Ponziani FR, Calvani R, Marini F, Biancolillo A, Coelho-Júnior HJ, Gervasoni J, Primiano A, Putignani L, Del Chierico F, et al. Gut Microbial, Inflammatory and Metabolic Signatures in Older People with Physical Frailty and Sarcopenia: Results from the BIOSPHERE Study. *Nutrients* (2020) 12:65. doi: 10.3390/nu12010065

203. Joossens M, Huys G, Cnockaert M, Preter VD, Verbeke K, Rutgeerts P, Vandamme P, Vermeire S. Dysbiosis of the faecal microbiota in patients with Crohn’s disease and their unaffected relatives. *Gut* (2011) 60:631–637. doi: 10.1136/gut.2010.223263

204. Ueda A, Shinkai S, Shiroma H, Taniguchi Y, Tsuchida S, Kariya T, Kawahara T, Kobayashi Y, Kohda N, Ushida K, et al. Identification of Faecalibacterium prausnitzii strains for gut microbiome-based intervention in Alzheimer’s-type dementia. *Cell Rep Med* (2021) 2:100398. doi: 10.1016/j.xcrm.2021.100398

205. Demirci M, Tokman HB, Uysal HK, Demiryas S, Karakullukcu A, Saribas S, Cokugras H, Kocazeybek BS. Reduced *Akkermansia muciniphila* and *Faecalibacterium prausnitzii* levels in the gut microbiota of children with allergic asthma. *Allergol Immunopathol (Madr)* (2019) 47:365–371. doi: 10.1016/j.aller.2018.12.009

206. Geravand M, Fallah P, Yaghoobi MH, Soleimanifar F, Farid M, Zinatizadeh N, Yaslianifard S. INVESTIGATION OF *ENTEROCOCCUS FAECALIS* POPULATION IN PATIENTS WITH POLYP AND COLORECTAL CANCER IN COMPARISON OF HEALTHY INDIVIDUALS. *Arq Gastroenterol* (2019) 56:141–145. doi: 10.1590/S0004-2803.201900000-28

207. Li W, Wu X, Hu X, Wang T, Liang S, Duan Y, Jin F, Qin B. Structural changes of gut microbiota in Parkinson’s disease and its correlation with clinical features. *Sci China Life Sci* (2017) 60:1223–1233. doi: 10.1007/s11427-016-9001-4

208. Bojović K, Ignjatović Đ -d ica, Soković Bajić S, Vojnović Milutinović D, Tomić M, Golić N, Tolinački M. Gut Microbiota Dysbiosis Associated With Altered Production of Short Chain

Fatty Acids in Children With Neurodevelopmental Disorders. *Front Cell Infect Microbiol* (2020) 10. doi: 10.3389/fcimb.2020.00223

209. Knoll RL, Forslund K, Kultima JR, Meyer CU, Kullmer U, Sunagawa S, Bork P, Gehring S. Gut microbiota differs between children with Inflammatory Bowel Disease and healthy siblings in taxonomic and functional composition: a metagenomic analysis. *Am J Physiol-Gastrointest Liver Physiol* (2017) 312:G327–G339. doi: 10.1152/ajpgi.00293.2016

210. Zhou Y, Zhi F. Lower Level of Bacteroides in the Gut Microbiota Is Associated with Inflammatory Bowel Disease: A Meta-Analysis. *BioMed Res Int* (2016) 2016:e5828959. doi: 10.1155/2016/5828959

211. Jakobsson HE, Abrahamsson TR, Jenmalm MC, Harris K, Quince C, Jernberg C, Björkstén B, Engstrand L, Andersson AF. Decreased gut microbiota diversity, delayed Bacteroidetes colonisation and reduced Th1 responses in infants delivered by Caesarean section. *Gut* (2014) 63:559–566. doi: 10.1136/gutjnl-2012-303249

212. Kovatcheva-Datchary P, Nilsson A, Akrami R, Lee YS, De Vadder F, Arora T, Hallen A, Martens E, Björck I, Bäckhed F. Dietary Fiber-Induced Improvement in Glucose Metabolism Is Associated with Increased Abundance of Prevotella. *Cell Metab* (2015) 22:971–982. doi: 10.1016/j.cmet.2015.10.001

213. Li J, Zhao F, Wang Y, Chen J, Tao J, Tian G, Wu S, Liu W, Cui Q, Geng B, et al. Gut microbiota dysbiosis contributes to the development of hypertension. *Microbiome* (2017) 5:14. doi: 10.1186/s40168-016-0222-x

214. Wen N-N, Sun L-W, Geng Q, Zheng G-H. Gut microbiota changes associated with frailty in older adults: A systematic review of observational studies. *World J Clin Cases* (2024) 12:6815–6825. doi: 10.12998/wjcc.v12.i35.6815

215. Frémont M, Coomans D, Massart S, De Meirleir K. High-throughput 16S rRNA gene sequencing reveals alterations of intestinal microbiota in myalgic encephalomyelitis/chronic fatigue syndrome patients. *Anaerobe* (2013) 22:50–56. doi: 10.1016/j.anaerobe.2013.06.002

216. Zuo K, Li J, Li K, Hu C, Gao Y, Chen M, Hu R, Liu Y, Chi H, Wang H, et al. Disordered gut microbiota and alterations in metabolic patterns are associated with atrial fibrillation. *GigaScience* (2019) 8:giz058. doi: 10.1093/gigascience/giz058

217. Xu Y, Wang Y, Li H, Dai Y, Chen D, Wang M, Jiang X, Huang Z, Yu H, Huang J, et al. Altered Fecal Microbiota Composition in Older Adults With Frailty. *Front Cell Infect Microbiol* (2021) 11: doi: 10.3389/fcimb.2021.696186

218. Ahrens AP, Hyötyläinen T, Petrone JR, Igelström K, George CD, Garrett TJ, Orešič M, Triplett EW, Ludvigsson J. Infant microbes and metabolites point to childhood neurodevelopmental disorders. *Cell* (2024) 187:1853-1873.e15. doi: 10.1016/j.cell.2024.02.035

219. F.S.Teixeira T, Grześkowiak ŁM, Salminen S, Laitinen K, Bressan J, Gouveia Peluzio M do C. Faecal levels of Bifidobacterium and Clostridium coccoides but not plasma lipopolysaccharide

are inversely related to insulin and HOMA index in women. *Clin Nutr* (2013) 32:1017–1022. doi: 10.1016/j.clnu.2013.02.008

220. Zhu L, Baker SS, Gill C, Liu W, Alkhouri R, Baker RD, Gill SR. Characterization of gut microbiomes in nonalcoholic steatohepatitis (NASH) patients: A connection between endogenous alcohol and NASH. *Hepatology* (2013) 57:601–609. doi: 10.1002/hep.26093

221. Victoria M, Elena V-DB, Amparo G-GN, María J-RA, Adriana G-V, Irene A-C, Alejandra Y-MM, Janeth B-B, María A-OG. Gut microbiota alterations in critically ill older patients: a multicenter study. *BMC Geriatr* (2022) 22:373. doi: 10.1186/s12877-022-02981-0

222. Wang L, Alammar N, Singh R, Nanavati J, Song Y, Chaudhary R, Mullin GE. Gut Microbial Dysbiosis in the Irritable Bowel Syndrome: A Systematic Review and Meta-Analysis of Case-Control Studies. *J Acad Nutr Diet* (2020) 120:565–586. doi: 10.1016/j.jand.2019.05.015

223. Bellocchi C, Fernández-Ochoa Á, Montanelli G, Vigone B, Santaniello A, Milani C, Quirantes-Piné R, Borrás-Linares I, Ventura M, Segura-Carrettero A, et al. Microbial and metabolic multi-omic correlations in systemic sclerosis patients. *Ann N Y Acad Sci* (2018) 1421:97–109. doi: 10.1111/nyas.13736

224. Murros KE, Huynh VA, Takala TM, Saris PEJ. Desulfovibrio Bacteria Are Associated With Parkinson’s Disease. *Front Cell Infect Microbiol* (2021) 11. doi: 10.3389/fcimb.2021.652617

225. Dinh DM, Ramadass B, Kattula D, Sarkar R, Braunstein P, Tai A, Wanke CA, Hassoun S, Kane AV, Naumova EN, et al. Longitudinal Analysis of the Intestinal Microbiota in Persistently Stunted Young Children in South India. *PLoS ONE* (2016) 11:e0155405. doi: 10.1371/journal.pone.0155405

226. Karlsson CLJ, Önnerfält J, Xu J, Molin G, Ahrné S, Thorngren-Jerneck K. The Microbiota of the Gut in Preschool Children With Normal and Excessive Body Weight. *Obesity* (2012) 20:2257–2261. doi: 10.1038/oby.2012.110

227. Png CW, Lindén SK, Gilshenan KS, Zoetendal EG, McSweeney CS, Sly LI, McGuckin MA, Florin THJ. Mucolytic Bacteria With Increased Prevalence in IBD Mucosa AugmentIn VitroUtilization of Mucin by Other Bacteria. *Off J Am Coll Gastroenterol ACG* (2010) 105:2420–2428. doi: 10.1038/ajg.2010.281

228. Santacruz A, Collado MC, García-Valdés L, Segura MT, Martín-Lagos JA, Anjos T, Martí-Romero M, Lopez RM, Florido J, Campoy C, et al. Gut microbiota composition is associated with body weight, weight gain and biochemical parameters in pregnant women. *Br J Nutr* (2010) 104:83–92. doi: 10.1017/S0007114510000176

229. Camara A, Konate S, Tidjani Alou M, Kodio A, Togo AH, Cortaredona S, Henrissat B, Thera MA, Doumbo OK, Raoult D, et al. Clinical evidence of the role of Methanobrevibacter smithii in severe acute malnutrition. *Sci Rep* (2021) 11:5426. doi: 10.1038/s41598-021-84641-8

230. Shoubridge AP, Carpenter L, Flynn E, Papanicolas LE, Collins J, Gordon D, Lynn DJ, Whitehead C, Leong LEX, Cations M, et al. Severe cognitive impairment is linked to a reduced gut microbiome capacity to synthesise immunomodulators, neurotransmitters, and amino acids

required for autophagy in residents of long-term aged care. (2023)2023.03.06.23286878. doi: 10.1101/2023.03.06.23286878

231. Sokol H, Leducq V, Aschard H, Pham H-P, Jegou S, Landman C, Cohen D, Liguori G, Bourrier A, Nion-Larmurier I, et al. Fungal microbiota dysbiosis in IBD. *Gut* (2017) 66:1039–1048. doi: 10.1136/gutjnl-2015-310746

232. Strati F, Cavalieri D, Albanese D, De Felice C, Donati C, Hayek J, Jousson O, Leoncini S, Renzi D, Calabrò A, et al. New evidences on the altered gut microbiota in autism spectrum disorders. *Microbiome* (2017) 5:24. doi: 10.1186/s40168-017-0242-1

233. Ling Z, Zhu M, Liu X, Shao L, Cheng Y, Yan X, Jiang R, Wu S. Fecal Fungal Dysbiosis in Chinese Patients With Alzheimer's Disease. *Front Cell Dev Biol* (2021) 8: doi: 10.3389/fcell.2020.631460

234. Gao R, Xia K, Wu M, Zhong H, Sun J, Zhu Y, Huang L, Wu X, Yin L, Yang R, et al. Alterations of Gut Mycobiota Profiles in Adenoma and Colorectal Cancer. *Front Cell Infect Microbiol* (2022) 12. doi: 10.3389/fcimb.2022.839435

235. Zou R, Wang Y, Duan M, Guo M, Zhang Q, Zheng H. Dysbiosis of Gut Fungal Microbiota in Children with Autism Spectrum Disorders. *J Autism Dev Disord* (2021) 51:267–275. doi: 10.1007/s10803-020-04543-y

236. Bridgman SL, Azad MB, Field CJ, Haqq AM, Becker AB, Mandhane PJ, Subbarao P, Turvey SE, Sears MR, Scott JA, et al. Fecal Short-Chain Fatty Acid Variations by Breastfeeding Status in Infants at 4 Months: Differences in Relative versus Absolute Concentrations. *Front Nutr* (2017) 4: doi: 10.3389/fnut.2017.00011

237. Kim KN, Yao Y, Ju SY. Short Chain Fatty Acids and Fecal Microbiota Abundance in Humans with Obesity: A Systematic Review and Meta-Analysis. *Nutrients* (2019) 11:2512. doi: 10.3390/nu11102512

238. Wu L, Han Y, Zheng Z, Peng G, Liu P, Yue S, Zhu S, Chen J, Lv H, Shao L, et al. Altered Gut Microbial Metabolites in Amnestic Mild Cognitive Impairment and Alzheimer's Disease: Signals in Host–Microbe Interplay. *Nutrients* (2021) 13:228. doi: 10.3390/nu13010228

239. Da Silva HE, Teterina A, Comelli EM, Taibi A, Arendt BM, Fischer SE, Lou W, Allard JP. Nonalcoholic fatty liver disease is associated with dysbiosis independent of body mass index and insulin resistance. *Sci Rep* (2018) 8:1466. doi: 10.1038/s41598-018-19753-9

240. Szczesniak O, Hestad KA, Hanssen JF, Rudi K. Isovaleric acid in stool correlates with human depression. *Nutr Neurosci* (2016) 19:279–283. doi: 10.1179/1476830515Y.0000000007

241. Gall GL, Guttula K, Kellingray L, Tett AJ, Hoopen R ten, Kemsley EK, Savva GM, Ibrahim A, Narbad A. Metabolite quantification of faecal extracts from colorectal cancer patients and healthy controls. *Oncotarget* (2018) 9:33278–33289. doi: 10.18632/oncotarget.26022

242. Kok CR, Brabec B, Chichlowski M, Harris CL, Moore N, Wampler JL, Vanderhoof J, Rose D, Hutkins R. Stool microbiome, pH and short/branched chain fatty acids in infants receiving

extensively hydrolyzed formula, amino acid formula, or human milk through two months of age. *BMC Microbiol* (2020) 20:337. doi: 10.1186/s12866-020-01991-5

243. Tricon S, Burdge GC, Kew S, Banerjee T, Russell JJ, Grimble RF, Williams CM, Calder PC, Yaqoob P. Effects of cis-9,trans-11 and trans-10,cis-12 conjugated linoleic acid on immune cell function in healthy humans. *Am J Clin Nutr* (2004) 80:1626–1633. doi: 10.1093/ajcn/80.6.1626

244. Racine NM, Watras AC, Carrel AL, Allen DB, McVean JJ, Clark RR, O'Brien AR, O'Shea M, Scott CE, Schoeller DA. Effect of conjugated linoleic acid on body fat accretion in overweight or obese children123. *Am J Clin Nutr* (2010) 91:1157–1164. doi: 10.3945/ajcn.2009.28404

245. Moloney F, Yeow T-P, Mullen A, Nolan JJ, Roche HM. Conjugated linoleic acid supplementation, insulin sensitivity, and lipoprotein metabolism in patients with type 2 diabetes mellitus123. *Am J Clin Nutr* (2004) 80:887–895. doi: 10.1093/ajcn/80.4.887

246. Graham SM, Arvela OM, Wise GA. Long-term neurologic consequences of nutritional vitamin B12 deficiency in infants. *J Pediatr* (1992) 121:710–714. doi: 10.1016/S0022-3476(05)81897-9

247. Kivipelto M, Annerbo S, Hultdin J, Bäckman L, Viitanen M, Fratiglioni L, Lökk J. Homocysteine and holo-transcobalamin and the risk of dementia and Alzheimers disease: a prospective study. *Eur J Neurol* (2009) 16:808–813. doi: 10.1111/j.1468-1331.2009.02590.x

248. Järvinen E, Ismail K, Muniandy M, Bogl LH, Heinonen S, Tummers M, Miettinen S, Kaprio J, Rissanen A, Ollikainen M, et al. Biotin-dependent functions in adiposity: a study of monozygotic twin pairs. *Int J Obes* (2016) 40:788–795. doi: 10.1038/ijo.2015.237

249. Shea MK, Booth SL, Massaro JM, Jacques PF, D'Agostino RB Sr, Dawson-Hughes B, Ordovas JM, O'Donnell CJ, Kathiresan S, Keaney JF Jr, et al. Vitamin K and Vitamin D Status: Associations with Inflammatory Markers in the Framingham Offspring Study. *Am J Epidemiol* (2008) 167:313–320. doi: 10.1093/aje/kwm306

250. Shea KM, Cushman M, Booth SL, Burke GL, Chen H, Kritchevsky SB. Associations between vitamin K status and haemostatic and inflammatory biomarkers in community-dwelling adults. *Thromb Haemost* (2014) 112:438–444. doi: 10.1160/TH13-12-1003

251. YILMAZ C, YUCA SA, YILMAZ N, BEKTAŞ MS, ÇAKSEN H. Intracranial Hemorrhage Due to Vitamin K Deficiency in Infants: A Clinical Study. *Int J Neurosci* (2009) 119:2250–2256. doi: 10.3109/00207450903170437

252. Pham VT, Lacroix C, Braegger CP, Chassard C. Lactate-utilizing community is associated with gut microbiota dysbiosis in colicky infants. *Sci Rep* (2017) 7:11176. doi: 10.1038/s41598-017-11509-1

253. Chassard C, Dapoigny M, Scott KP, Crouzet L, Del'homme C, Marquet P, Martin JC, Pickering G, Ardid D, Eschalier A, et al. Functional dysbiosis within the gut microbiota of patients with constipated-irritable bowel syndrome. *Aliment Pharmacol Ther* (2012) 35:828–838. doi: 10.1111/j.1365-2036.2012.05007.x

254. An R, Wilms E, Logtenberg MJ, van Trijp MPH, Schols HA, Masclee AAM, Smidt H, Jonkers DMAE, Zoetendal EG. In vitro metabolic capacity of carbohydrate degradation by intestinal microbiota of adults and pre-frail elderly. *ISME Commun* (2021) 1:1–12. doi: 10.1038/s43705-021-00065-5

255. Jiang T, Suarez FL, Levitt MD, Nelson SE, Ziegler EE. Gas production by feces of infants. *J Pediatr Gastroenterol Nutr* (2001) 32:534–541. doi: 10.1097/00005176-200105000-00009

256. Yang X, Xiu W-B, Wang J-X, Li L-P, He C, Gao C-P. CO2 Is Beneficial to Gut Microbiota Homeostasis during Colonoscopy: Randomized Controlled Trial. *J Clin Med* (2022) 11:5281. doi: 10.3390/jcm11185281

257. Yamano H, Yoshikawa K, Kimura T, Yamamoto E, Harada E, Kudou T, Katou R, Hayashi Y, Satou K. Carbon dioxide insufflation for colonoscopy: evaluation of gas volume, abdominal pain, examination time and transcutaneous partial CO2 pressure. *J Gastroenterol* (2010) 45:1235–1240. doi: 10.1007/s00535-010-0286-5

258. Jangi S, Gandhi R, Cox LM, Li N, von Glehn F, Yan R, Patel B, Mazzola MA, Liu S, Glanz BL, et al. Alterations of the human gut microbiome in multiple sclerosis. *Nat Commun* (2016) 7:12015. doi: 10.1038/ncomms12015

259. Soares ACF, Tahan S, Fagundes-Neto U, de Morais MB. [Breath methane in children with chronic constipation]. *Arq Gastroenterol* (2002) 39:66–72. doi: 10.1590/s0004-28032002000100012

260. Piragine E, Malanima MA, Lucenteforte E, Martelli A, Calderone V. Circulating Levels of Hydrogen Sulfide (H2S) in Patients with Age-Related Diseases: A Systematic Review and Meta-Analysis. *Biomolecules* (2023) 13:1023. doi: 10.3390/biom13071023

261. Lu Q, Chen J, Jiang L, Geng T, Tian S, Liao Y, Yang K, Zheng Y, He M, Tang H, et al. Gut microbiota-derived secondary bile acids, bile acids receptor polymorphisms, and risk of cardiovascular disease in individuals with newly diagnosed type 2 diabetes: a cohort study. *Am J Clin Nutr* (2024) 119:324–332. doi: 10.1016/j.ajcnut.2023.08.023

262. Li Y, Zhang D, He Y, Chen C, Song C, Zhao Y, Bai Y, Wang Y, Pu J, Chen J, et al. Investigation of novel metabolites potentially involved in the pathogenesis of coronary heart disease using a UHPLC-QTOF/MS-based metabolomics approach. *Sci Rep* (2017) 7:15357. doi: 10.1038/s41598-017-15737-3

263. Huang W-K, Hsu H-C, Liu J-R, Yang T-S, Chen J-S, Chang JW-C, Lin Y-C, Yu K-H, Kuo C-F, See L-C. The Association of Ursodeoxycholic Acid Use With Colorectal Cancer Risk: A Nationwide Cohort Study. *Medicine (Baltimore)* (2016) 95:e2980. doi: 10.1097/MD.0000000000002980

264. Hill DR, Buck RH. Infants Fed Breastmilk or 2′-FL Supplemented Formula Have Similar Systemic Levels of Microbiota-Derived Secondary Bile Acids. *Nutrients* (2023) 15:2339. doi: 10.3390/nu15102339

265. Kean IRL, Wagner J, Wijeyesekera A, De Goffau M, Thurston S, Clark JA, White DK, Ridout J, Agrawal S, Kayani R, et al. Profiling gut microbiota and bile acid metabolism in critically ill children. *Sci Rep* (2022) 12:10432. doi: 10.1038/s41598-022-13640-0

266. Pan J-X, Xia J-J, Deng F-L, Liang W-W, Wu J, Yin B-M, Dong M-X, Chen J-J, Ye F, Wang H-Y, et al. Diagnosis of major depressive disorder based on changes in multiple plasma neurotransmitters: a targeted metabolomics study. *Transl Psychiatry* (2018) 8:1–10. doi: 10.1038/s41398-018-0183-x

267. Manca R, De Marco M, Soininen H, Ruffini L, Venneri A. Changes in neurotransmitter-related functional connectivity along the Alzheimer's disease continuum. *Brain Commun* (2025)fcaf008. doi: 10.1093/braincomms/fcaf008

268. Abdulamir HA, Abdul-Rasheed OF, Abdulghani EA. Serotonin and serotonin transporter levels in autistic children. *Saudi Med J* (2018) 39:487–494. doi: 10.15537/smj.2018.5.21751

269. Song Q, Hwang C-L, Li Y, Wang J, Park J, Lee SM, Sun Z, Sun J, Xia Y, Nieto N, et al. Gut-derived ammonia contributes to alcohol-related fatty liver development via facilitating ethanol metabolism and provoking ATF4-dependent *de novo* lipogenesis activation. *Metabolism* (2024) 151:155740. doi: 10.1016/j.metabol.2023.155740

270. Fisman M, Ball M, Blume W. Hyperammonemia and Alzheimer's Disease. *J Am Geriatr Soc* (1989) 37:1102–1102. doi: 10.1111/j.1532-5415.1989.tb06935.x

271. Ozanne B, Nelson J, Cousineau J, Lambert M, Phan V, Mitchell G, Alvarez F, Ducruet T, Jouvet P. Threshold for toxicity from hyperammonemia in critically ill children. *J Hepatol* (2012) 56:123–128. doi: 10.1016/j.jhep.2011.03.021

272. Ikematsu N, Kashiwagi M, Hara K, Waters B, Matsusue A, Takayama M, Kubo S. Organ distribution of endogenous p-cresol in hemodialysis patients. *J Med Invest* (2019) 66:81–85. doi: 10.2152/jmi.66.81

273. Gabriele S, Sacco R, Cerullo S, Neri C, Urbani A, Tripi G, Malvy J, Barthelemy C, Bonnet-Brihault F, Persico AM. Urinary p-cresol is elevated in young French children with autism spectrum disorder: a replication study. *Biomarkers* (2014) 19:463–470. doi: 10.3109/1354750X.2014.936911

274. Banerjee T, Meyer TW, Shafi T, Hostetter TH, Melamed M, Zhu Y, Powe NR. Free and total p-cresol sulfate levels and infectious hospitalizations in hemodialysis patients in CHOICE and HEMO. *Medicine (Baltimore)* (2017) 96:e5799. doi: 10.1097/MD.0000000000005799

275. Alexeev EE, Lanis JM, Kao DJ, Campbell EL, Kelly CJ, Battista KD, Gerich ME, Jenkins BR, Walk ST, Kominsky DJ, et al. Microbiota-Derived Indole Metabolites Promote Human and Murine Intestinal Homeostasis through Regulation of Interleukin-10 Receptor. *Am J Pathol* (2018) 188:1183–1194. doi: 10.1016/j.ajpath.2018.01.011

276. Ehrlich AM, Pacheco AR, Henrick BM, Taft D, Xu G, Huda MN, Mishchuk D, Goodson ML, Slupsky C, Barile D, et al. Indole-3-lactic acid associated with Bifidobacterium-dominated

microbiota significantly decreases inflammation in intestinal epithelial cells. *BMC Microbiol* (2020) 20:357. doi: 10.1186/s12866-020-02023-y

277. Hietbrink F, Besselink MGH, Renooij W, de Smet MBM, Draisma A, van der Hoeven H, Pickkers P. SYSTEMIC INFLAMMATION INCREASES INTESTINAL PERMEABILITY DURING EXPERIMENTAL HUMAN ENDOTOXEMIA. *Shock* (2009) 32:374–378. doi: 10.1097/SHK.0b013e3181a2bcd6

278. Vatanen T, Kostic AD, d'Hennezel E, Siljander H, Franzosa EA, Yassour M, Kolde R, Vlamakis H, Arthur TD, Hämäläinen A-M, et al. Variation in Microbiome LPS Immunogenicity Contributes to Autoimmunity in Humans. *Cell* (2016) 165:842–853. doi: 10.1016/j.cell.2016.04.007
